# Markers for blood-brain barrier integrity: how appropriate is Evans blue in the twenty-first century and what are the alternatives?

**DOI:** 10.3389/fnins.2015.00385

**Published:** 2015-10-29

**Authors:** Norman R. Saunders, Katarzyna M. Dziegielewska, Kjeld Møllgård, Mark D. Habgood

**Affiliations:** ^1^Laboratory of Developmental Neurobiology and Neurotrauma, Department of Pharmacology and Therapeutics, University of MelbourneParkville, VIC, Australia; ^2^Department of Cellular and Molecular Medicine, University of CopenhagenCopenhagen, Denmark

**Keywords:** blood-brain barrier, embryo, fetus, newborn, permeability, tight junctions

## Abstract

In recent years there has been a resurgence of interest in brain barriers and various roles their intrinsic mechanisms may play in neurological disorders. Such studies require suitable models and markers to demonstrate integrity and functional changes at the interfaces between blood, brain, and cerebrospinal fluid. Studies of brain barrier mechanisms and measurements of plasma volume using dyes have a long-standing history, dating back to the late nineteenth-century. Their use in blood-brain barrier studies continues in spite of their known serious limitations in *in vivo* applications. These were well known when first introduced, but seem to have been forgotten since. Understanding these limitations is important because Evans blue is still the most commonly used marker of brain barrier integrity and those using it seem oblivious to problems arising from its *in vivo* application. The introduction of HRP in the mid twentieth-century was an important advance because its reaction product can be visualized at the electron microscopical level, but it also has limitations. Advantages and disadvantages of these markers will be discussed together with a critical evaluation of alternative approaches. There is no single marker suitable for all purposes. A combination of different sized, visualizable dextrans and radiolabeled molecules currently seems to be the most appropriate approach for qualitative and quantitative assessment of barrier integrity.

## Introduction

**Figure 1:**
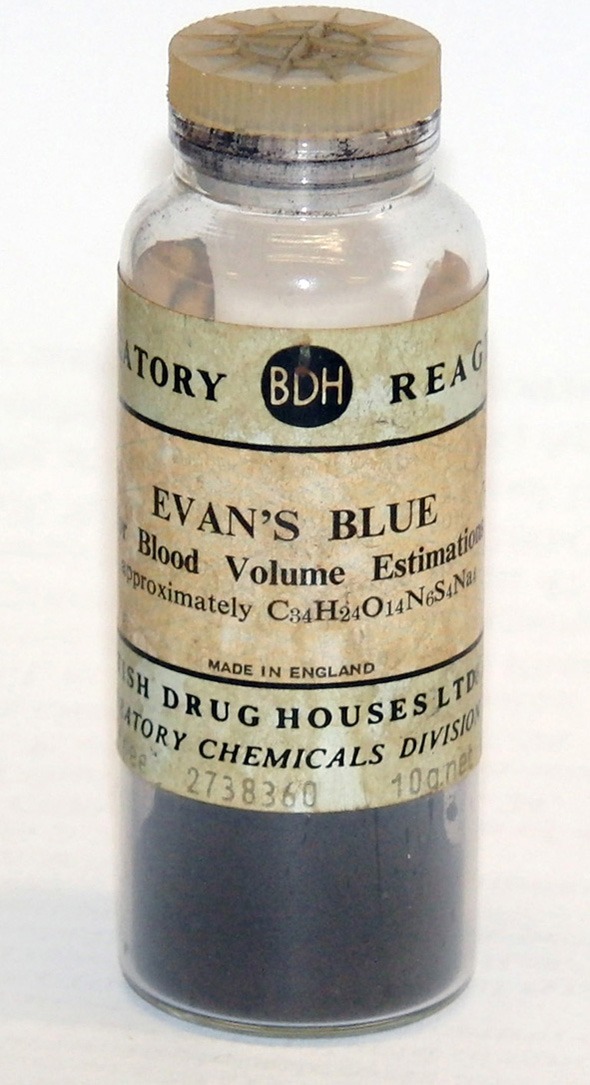
**Evans blue**. As used at University College London, Department of Physiology circa 1960 for *in vivo* plasma volume estimation.

**Figure 2:**
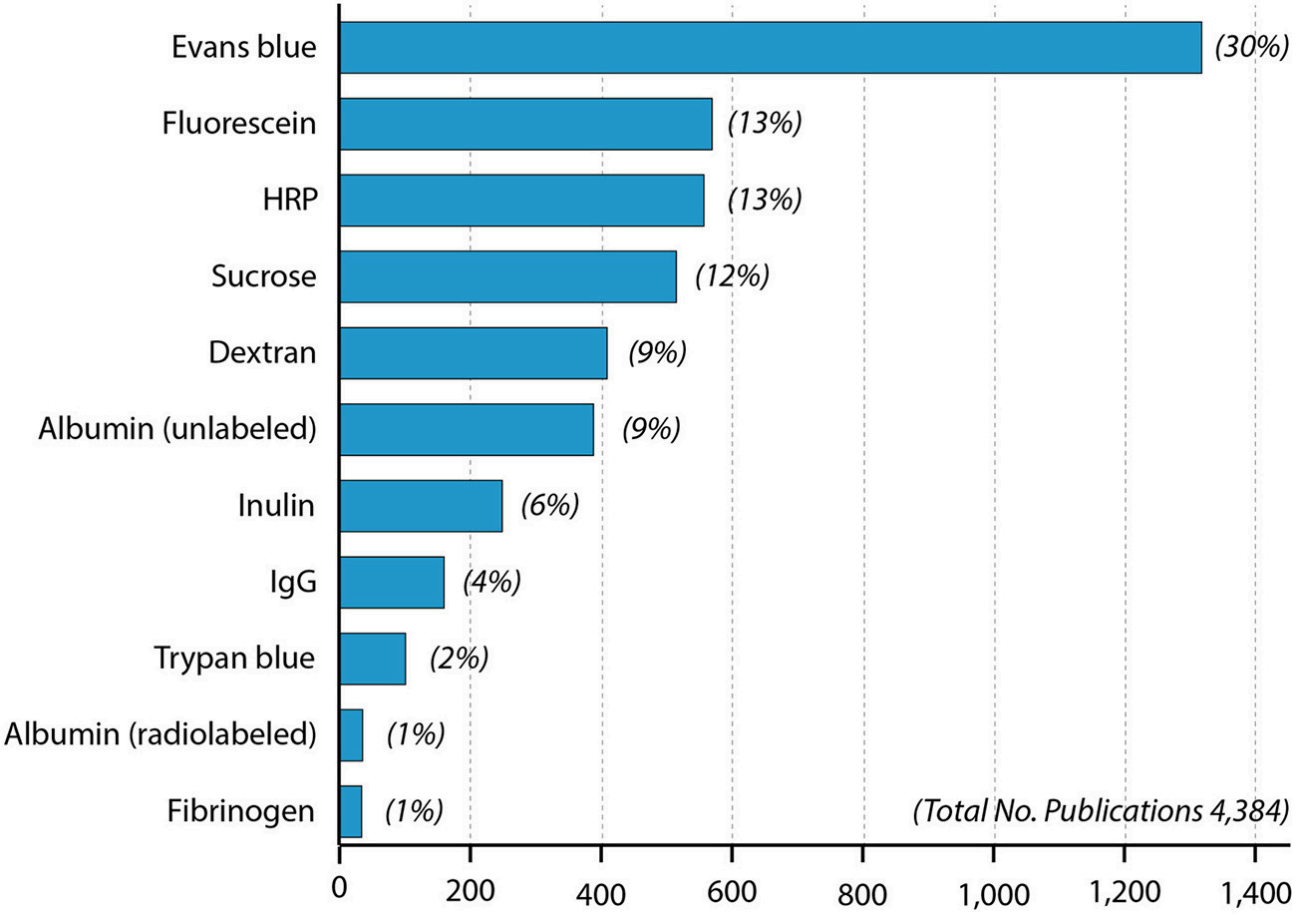
**Papers in PubMed using different blood-brain barrier markers since 1953**. One curiosity is that radiolabeled albumin was used more than a decade (Ashkenazy and Crawley, 1953) before the first use of Evans blue (Rössner and Temple, 1966) but has only been infrequently used since then compared to Evans blue.

**Figure 3:**
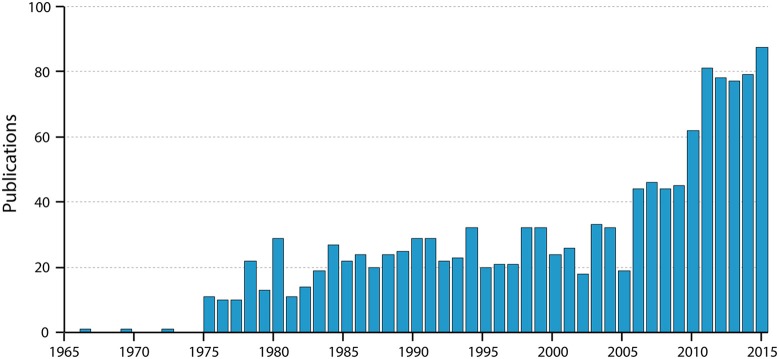
**Numbers of papers by year listed in PubMed for “blood-brain barrier Evans blue.”** Note the very steep increase in the past 10 years; note also that the value for 2015 is for only 9 months of the year. Thus, the use of Evans blue is still clearly increasing substantially.

The realization that brain barriers may play a critical role in a wide range of neurological disorders prompted a renewed interest in studies of their function and integrity (Saunders et al., 2008). Such studies require suitable models and especially markers to demonstrate integrity of the interfaces between the blood, the brain and the cerebrospinal fluid, CSF. Similarly markers are also required for the study of barriers in the developing brain and in its evolution, which are the main focus of the Frontiers Topic “Ontogeny and Phylogeny of Brain Barrier Mechanisms.” Dyes have a venerable history dating back to the end of the nineteenth-century, in studies of brain barrier mechanisms in both the developing and adult brain, although many ascribe incorrectly the first use of dyes for this purpose to Ehrlich in the mid nineteenth-century or to Goldmann (1909, 1913), in the early twentieth century (see Saunders et al., 2014). In times when there were no alternatives it seems reasonable that dyes should have been used as markers for brain barrier integrity, similar to their use in cardiovascular studies for measurement of plasma volume (Dawson et al., 1920). Particularly in the latter field scientists were well aware of the limitations of dyes and as soon as more satisfactory alternatives became available, notably radiolabeled proteins such as albumin, dyes were rapidly abandoned. In striking contrast, the use of dyes remained widespread in blood-brain barrier field in spite of their limitations, which have been well described since the mid twentieth-century. One dye in particular, Evans blue (Figure 1) is still the most commonly used marker of brain barrier integrity (Figure 2) and its use has increased substantially in recent years (Figure 3). Limitations of Evans blue as applied to studies of brain barriers, as well as of other dyes, will be reviewed here together with their properties. Another commonly used marker for brain barrier integrity is HRP (introduced in mid twentieth-century for electron microscopy studies by Straus (1959), Reese and Karnovsky (1967), and Brightman and Reese (1969) will also be discussed in this review.

The characteristics of an “ideal” marker for evaluating brain barrier integrity are that it should be metabolically inert, non-toxic, not bound to other molecules such as proteins in plasma or tissues, be available in a range of molecular sizes, have the ability to be visualized in the range from the naked eye to the electron microscopical level and be reliably quantifiable. The only merit of Evans blue dye is that is visible to the unaided eye and can thus give a gross indication of its distribution. However, it fails on all other counts. Thus, studies using only Evans blue should no longer be published, as there are much better markers available.

There is no single marker currently available that fulfills all of the specified criteria, however different molecular sized dextrans labeled with either biotin or a fluorescent tag come close. Their main limitation is probably that they are tedious to quantify; for this purpose it is better to use radiolabeled markers, which are available in a wide range of molecular sizes but have the disadvantage that currently they cannot be visualized with sufficient resolution. Thus, a combination of different dextrans and radiolabeled molecules is currently the most appropriate approach to assessing barrier integrity in the developing or pathological brain.

## Use of dyes for estimation of blood volume

A summary of this field is included because it was the studies of the properties of dyes in circulating blood as established by cardiovascular physiologists, which provided much of the later justification for their use by blood-brain barrier biologists. The significance of this is that in the early blood-brain barrier studies it was not well appreciated that the dyes being used, such as trypan blue, bind to proteins in plasma, although there were some early hints that this might be the case (see review by Saunders et al., 2014). The earliest dye dilution estimation of plasma volume appears to be Keith et al. (1915). These authors highlighted the unsatisfactory, unreliable and potentially injurious methods previously used, which included bleeding to death (of convicts) and inhalation of, or injection of blood samples saturated with carbon monoxide. They proposed the alternative method of a non-toxic, slowly absorbable dye that remained in the circulation and suggested a colorimetric determination of its concentration in plasma by comparison with a mixture of dye and serum. On the advice of Dr. Evans in the Anatomical Department (University of California, San Francisco) Keith et al. (1915) used *vital red*, which Evans supplied. This resulted in several extensive published studies in dogs and humans. In later work in dogs *brilliant vital red* was used (Hooper et al., 1920; Smith, 1920). This dye had a number of disadvantages including difficulty in identifying when haemolysis had occurred in the blood samples. Dawson et al. (1920) investigated a series of dyes and concluded that the *blue azo* dye (T-1824) was slightly superior to all the *vital red* dyes tested. T-1824 was one of a large series of azo dyes developed in the late nineteenth early twentieth centuries by the German dye industry. T-1824 was its industrial code number. It is so named because it can be synthesized by coupling together one mole of diazotized o-toluidine and two moles of τ-amino-8-naphthol-2, 4-disulfonic acid (Allen and Orahovats, 1950).

One of the co-authors of Dawson et al. (1920) was Evans. The name of the dye was much later changed from T-1824 to Evans blue in recognition of his contributions to the use of dyes in animal studies; this designation has been suggested to have been introduced by the Eastman Kodak Company, which marketed the dye with this name (Cooksey, 2013). However, this name was deplored by Gregersen and Stewart (1938) and Gregersen's group persisted with the original designation of T-1824 right up to their last publication in the field (Gregersen and Rawson, 1959). In the rest of the cardiovascular field the designation T-1824 is rarely used unless in combination with Evans blue and is not used at all in the blood-brain barrier field. To avoid confusion, when discussing early literature in which only the designation T-1824 has been used, we have retained it in the form: T-1824 (Evans blue).

A key criterion for a suitable dye was that it remained in the circulation at a constant level for several minutes (optimum was considered to be 4 min). Many of the dyes were found to be unsuitable because they disappeared from the blood rapidly, in some cases via the kidneys. The authors had no explanation for this difference compared to the azo dyes like T-1824 (Evans blue), which was relatively stable in the blood. It seems most likely that the excreted dyes, in contrast to Evans blue, were not bound to plasma proteins, see below. The conclusion of Dawson and his colleagues was that T-1824 (Evans blue) was marginally superior to previously used vital red dyes and others that they tested for measurement of plasma (blood) volume because colorimetric estimation was both easier and more accurate.

One of the first uses of T-1824 (Evans blue) in humans appears to have been by Gregersen and Stewart (1938). Prior to its introduction various red dyes or trypan blue were used. One advantage of T-1824 (Evans blue) was that much smaller concentrations could be estimated; thus some clinicians judged it to be superior to trypan blue because the large amount of that dye required for plasma volume estimations gave the patients a blue tinge, thus adding a cyanotic color to their already rather sickly appearance (Gregersen, 1938). The use of T-1824 (Evans blue) for estimation of plasma volume was extensively studied by Gregersen and colleagues over the period from 1933 to 1956. Gregersen (1938) in particular seems to have been preoccupied with the need to develop more accurate colorimetric methods than those of Keith et al. (1915) and modified by Smith (1920) and by Dawson et al. (1920). This was prompted by his initial studies undertaken at Harvard Medical School on the effects of thirst and reduced salivary flow on plasma volume in which he found these methods to give results that were of “questionable accuracy” (Gregersen, 1932). With a fortitude that would probably not be tolerated by modern grant agencies, he spent the next 5 years improving the method, with a particular focus on colorimetric methods used for measuring concentrations of vital red dyes or T-1824 in plasma. He concluded that T-1824 (Evans blue) was a substantially better dye for plasma volume measurements partly because much lower concentrations were required but also because haemolysis was much less of a problem for colorimetric measurements. Gregersen and colleagues at Columbia University published extensively on the evaluation and use of T-1824 (Evans blue) with comparisons with radioisotopic methods once these were introduced (reviewed in Gregersen and Rawson, 1959). Since that time T-1824 (Evans blue) has largely been replaced by radio-iodinated human serum albumin for indicator dilution estimations of plasma volume (Margouleff, 2013). This is in contrast to the persistence in the use of Evans blue in studies of blood-barrier barrier integrity to the present time; this is in spite of a similar availability of better methods for many years, as discussed below. One paper from the Columbia group of particular relevance for blood-brain barrier studies is that of Rawson (1943) not least because it has frequently been mis-cited. It provided a detailed analysis of the concentration dependence of binding of Evans blue to human albumin, but also provided evidence of binding to globulins. Because of the importance of this paper for blood brain barrier permeability it will be considered in some detail in a separate section on dye binding to plasma proteins, below.

## Evans blue for assessment of blood-brain barrier integrity

One of the earliest uses of Evans blue for blood-brain barrier experiments appears to be that of Rössner and Temple (1966); these authors cite Bauer et al. (1956) as having used Evans blue, although Bauer et al. (1956) actually used Geigy Blau, the properties of which seem to be poorly researched (Gregersen and Rawson, 1959). Rössner and Temple (1966) were primarily concerned with efforts to develop a method for measuring Evans blue in tissue for which purpose they used about 300 rats and 50 guinea pigs. They do not appear to have considered the appropriate concentration to use or in what form the dye was present, although they do appear to have been aware of at least some of the papers published by Gregersen and colleagues. They used a large amount of dye (1 ml, 2% Evans blue per 100 g body weight) compared to the Gregersen group (e.g., 0.005–0.01 ml/100 g body weight, Gregersen and Stewart, 1938). It is unclear why such a large amount of dye was used by Rössner and Temple (1966) and in most subsequent studies by others, given that an important reason for using Evans blue for blood volume studies was that a much smaller amount of dye was required (Gregersen, 1938). Possibly it followed on from the protocols used for many years from the original experiments of Goldmann (1913) in which trypan blue was used to study blood-brain barrier permeability. Goldmann (1913) injected cats intravenously with 30–50 ml 1% trypan blue. In more recent times dye concentrations have usually been 2–4% but in lesser volumes. It is frequently stated in blood-brain barrier studies that Evans blue binds rapidly, tightly and exclusively to plasma albumin (Reeve, 1957; Stoelinga and van Munster, 1967; Wolman et al., 1981; Manaenko et al., 2011; Yen et al., 2013). However, this is not the case as will be considered below. The problems of using Evans blue as a marker for blood-brain barrier dysfunction can be summarized as (i) the likelihood of a substantial amount of free dye being present in an animal following the amounts injected, (ii) lack of specific binding to albumin (although this is widely believed, to the extent that many authors assume that the concentration of Evans blue in the brain is synonymous with penetration of albumin across a disrupted blood-brain barrier), (iii) injection of dye dissolved in “physiological saline” or other physiological solution, which have been suggested to affect the structure of the dye, (iv) there is evidence that Evans blue binds to tissues. (v) attempts to make quantitative assessments of damage to the blood-brain barrier in the brain are confounded by problems with the various spectroscopic methods that have been used to estimate the amount dye in brain tissue (not to mention the uncertainty about how much dye is bound to albumin and other plasma proteins, how much to brain tissue and how much may be free) (vi) *in vivo* potential lethal toxicity. Each of these problems is considered in some detail in the next sections of this review.

### (I) binding of evans blue (T-1824) and other dyes to plasma proteins: how much free dye is there?

Ehrlich (1885) suggested that the reason why some of the dyes, which he studied in animals, did not appear in the urine was that they might be bound to plasma albumin. This seems to have been overlooked by the blood-brain barrier field until the observations of Tschirgi (1950) on trypan blue and its exclusion from the brain. He compared the lack of staining of the brain when trypan blue was injected intravenously with the intense staining of brain that occurred when the same concentration (0.2%) of the dye was injected dissolved in Tyrode solution. He suggested that the lack of staining of the brain was due to binding of the dye to plasma albumin, which he confirmed by adding bovine albumin to the Tyrode-dye solution prior to injection. The earliest suggestion that Evans blue and other related dyes may bind to proteins in the blood appears to have been made by Gregersen and Rawson (1943) and was studied in detail by Rawson (1943) and by Allen and Orahovats (1950). Rawson (1943) used the electrophoresis method of Tiselius (1937) to study the binding of T-1824 (Evans blue) and structurally related diazo dyes to proteins in human plasma and to investigate the binding capacity of human albumin for T-1824 (Evans blue), trypan blue (T-1826), Niagara sky blue 6B and Niagara sky blue. In Rawson's (1943) experiments at low concentrations (0.004% or less) the dyes were wholly bound by albumin in either serum or plasma. But at higher concentrations this was no longer the case. Rawson (1943) estimated the binding capacity of human albumin as 8–14 moles of T-1824 (Evans blue); Rawson also found evidence for binding of the dye to globulins at higher concentrations. From their measurements of T-1824 (Evans blue) binding to albumin Allen and Orahovats (1950) showed that with an injection of 4 ml of 0.43% of the dye in a plasma volume and albumin concentration corresponding to that *in vivo* there would be measureable free dye (see Table 1).

**Table 1:** **Effect of albumin (Alb) molar concentration on binding of Evans blue dye (T-1824)**.

**Albumin Molar conc.**	**T-1824 Molar conc.**	**Unbound T-1824 Molar conc.**	**T-1824:Alb Molar ratio**	**Unbound, T-1824(%)**
1.23E-04	3.50E-03	2.13E-03	28.5	60.9
3.77E-04	1.76E-03	5.42E-06	4.7	0.31
5.03E-04	8.80E-04	1.40E-06	1.7	0.16
5.77E-04	3.52E-04	4.37E-07	0.61	0.12
6.04E-04	1.76E-04	2.00E-07	0.29	0.11
6.27E-04	1.76E-05	1.91E-08	0.03	0.11

Data in the first three columns are from the study of Allen and Orahovats (1950) in which 4 ml of a 0.43% solution of T-1824 was mixed with different volumes of a 4.4% albumin solution. At a ratio of 28.5 moles of T-1824 per mole of albumin, only around 40% of the T-1824 is actually bound to albumin (i.e., each albumin molecule binds a maximum of around 10 molecules of T-1824). Note that at all molar ratios less than this maximum binding capacity, a small proportion of T-1824 (0.11–0.31%) remains free in solution unbound to albumin.

Most studies using Evans blue to assess brain barrier integrity have used 2% or 4% solutions (e.g., Petito, 1979; Wolman et al., 1981; Abraham et al., 1996; Chen et al., 1996) thus at least when first entering the bloodstream a proportion of the dye will be free. An important aspect of Rawson's (1943) study and that of Allen and Orahovats (1950), which is almost universally ignored, is that as the dye concentration was increased an increasing proportion of the dye was free (i.e., not bound to albumin).

In blood-brain barrier experiments the volume injected is usually given, but rarely the size of the animals used, so it is often not possible to calculate the likely plasma concentration of the dye. However, a few papers do give the relevant information. Kaya and Ahishali (2011) published a methodological review of various blood-brain barrier integrity markers including Evans blue. In their description they indicate the use of 2% Evans blue dissolved in “physiological” saline solution administered intravenously at a dose of 4 ml per Kg body weight of animal. From this information the plasma concentration can be calculated assuming that all of the injected dye mixes with plasma before any significant losses, a haematocrit of 45% and a blood volume of 8% body weight for different species. This gives a dye concentration in plasma of 1.82 mg/ml or 0.18%. In rats the blood volume has been estimated as 6.44 ml/100 g (Lee and Blaufox, 1985) and the albumin concentration around 2250 mg/100 ml or 22.5 mg/ml (Dziegielewska et al., 1981). Thus, in this species, the plasma dye concentration would be 2.17 mg/ml (0.217%) at an Evans blue: albumin molar ratio of 6.52.

Spigelman et al. (1983) used 0.5 ml of a 2% Evans blue simultaneously with sodium fluorescein and ^99m^Tc-DTPA injected i.v. into 200–250 g rats; as all of these bind to plasma proteins it is likely there might have been some interference with the binding of Evans blue to albumin. However, assuming that this was not significant, the plasma concentration of Evans blue can be calculated as 1.12 mg/ml (0.11%) at an Evans blue: albumin molar ratio of 3.37.

Using an *in vitro* test of albumin concentration on the binding of Evans blue to cellophane, Rawson (1943) estimated a maximum binding capacity of less than 14 molecules of Evans blue per molecule of albumin. The data from Allen and Orahovats (1950) investigating the effect of albumin concentration on binding of Evans blue suggests a maximum binding capacity of 10 molecules of Evans blue per molecule of albumin (see Table 1). In both the Kaya and Ahishali (2011) and Spigelman et al. (1983) studies, the plasma concentration of Evans blue would probably not have exceeded the theoretical maximum binding capacity of albumin. However, an important point to note from the Allen and Orahovats (1950) study is that even at very low Evans blue: albumin ratios (< 1.0) a small proportion of Evans blue always remains unbound to albumin (see Table 1).

Moos and Møllgård (1993) re-investigated the problem of free dye following intraperitoneal or subcutaneous injections of different amounts of either trypan blue or Evans blue in mice. For concentrations of Evans blue of 1 or 2% in 0.9% saline (0.08 ml/20 g body weight) they found spectrophotometric evidence of substantial amounts of free dye in plasma at 30 min and 6 h following injection. For example using a 2% compared to 0.5% solution of Evans blue there was about 70% more free dye at 6 h after injection. On examining the brains of mice injected with this higher concentration of Evans blue, the dye was detected intraneuronally at several sites in the brain. Moos and Møllgård (1993) suggested a number of pathological mechanisms by which free dye might have entered the brain in their experiments and those of others.

Thus, it seems likely that there would be measureable amounts of free dye when concentrations of 2 or 4% were used, particularly in the first minutes after injection. That dye would be expected to rapidly enter the extracellular space of many tissues and bind to cells and matrix that have a higher affinity for Evans blue than the proteins in plasma.

### (ii) evans blue does not bind exclusively to albumin in plasma

**Figure 4:**
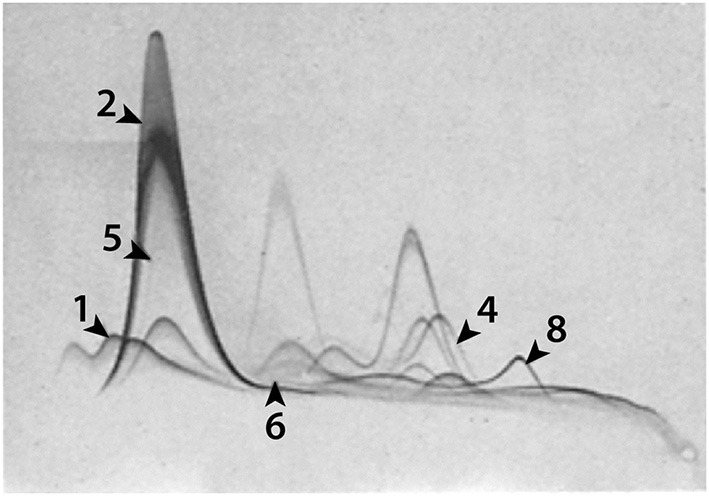
**Evans blue binding to 1, α_1_-lipoprotein; 2, albumin; 4, hemopexin; 5, prealbumin; 6, α_1_X and 8, transferrin**. Identified by anodal shifts. From Figure 3 in Emmett et al. (1985).

It is widely claimed that Evan blue binds tightly and exclusively to plasma albumin and that its visualization and/or quantitation can be used to define increases in blood brain barrier permeability to albumin (Wolman et al., 1981; Kitagawa et al., 1991; Nagaraja et al., 2008). As evidence some authors refer to Rawson (1943) but in most cases they simply refer to an earlier paper making this claim (e.g., Kang et al., 2013 refer to Spigelman et al., 1983 who do not actually mention Evans blue binding to albumin). Consideration of the literature on use of Evans blue for plasma volume measurements shows that such a claim is not supported by the experimental evidence. There are important species differences in the specificity and tightness of the binding and of particular importance is the fact that the binding of the dye to albumin (and other proteins, including tissue proteins) is a reversible equilibrium. Most of the original studies of Evans blue as a plasma marker were carried out in humans and dogs, species where the dye binding to albumin appears to be the strongest (Alle et al., 1953; Reeve, 1957). However, even in these species there were significant discrepancies in plasma volume measurements made with Evans blue (assumed to be bound to albumin) and with I^125^-albumin, suggesting that not all of the injected dye became bound to protein in the blood; thus “Evans blue-albumin” was found to have a larger distribution volume than I^125^-albumin (Carvalho, 1989). In some species e.g., rabbits, the discrepancy was even larger (Zizza and Reeve, 1958). The most cited study of Evans blue binding to albumin is that of Rawson (1943) who examined the binding to human albumin at different dye concentrations *in vitro* (not *in vivo* as stated by some authors; e.g., Uyama et al., 1988). However, Rawson used human albumin for which the binding of Evans blue is much tighter than for many animal species including rats (Alle et al., 1953; Emmett et al., 1985); at the concentrations used the dye would have been bound to other proteins in plasma (Rawson, 1943; LeVeen and Fishman, 1947; Emmett et al., 1985). This can be deduced from studies showing that Evans blue can bind to other proteins in plasma including globulins (LeVeen and Fishman, 1947), the post-albumin fraction (Linder and Heinle, 1982) as well as α_1_-1ipoprotein, hemopexin, and transferrin (Emmett et al., 1985). This is illustrated in Figure 4, where the authors used the elegant but little applied technique of crossed immunoeletrophoresis originally devised by Laurell (1965). The binding to plasma proteins in commonly used experimental animal species appears to be less tight than for human albumin (Alle et al., 1953; Emmett et al., 1985) and at the concentrations of the injected dye the binding capacity of albumin is likely to be exceeded, as discussed in the previous paragraph. As long ago as 1982 Linder and Heine concluded, “at the concentrations used by many investigators areas dyed by Evans blue may not be equated with the presence of diffusible protein-dye complexes.” This is a good, if neglected, summary of the problem. Because there is good evidence, as outlined above that Evans blue does not bind exclusively to albumin, its detection cannot be equated with the presence of albumin, especially as albumin in many cases is much better visualized using immunohistochemistry.

### (iii) appropriate carrier solutions for evans blue

According to Gregersen and Stewart (1938) Evans blue is not stable in “saline” solutions unless some protein is also present. They do not present any evidence for this, but given their extensive experience of working with the dye it seems appropriate to take note of this possible concern. Gregersen's group generally appears to have used water as the vehicle.

Some of the earlier studies, particularly those in which Evans blue was used to estimate plasma volume (e.g., Carvalho, 1989) the dye was mixed with plasma before injection. However, most if not all of the many studies of blood-brain barrier integrity published over the past 60 years appear to have used a “physiological solution” as the carrier (e.g., Ribatti et al., 1993; Kaya and Ahishali, 2011; Manaenko et al., 2011; Jiang et al., 2014) and in some there is no mention of the solution in which the Evans blue was dissolved (Kitagawa et al., 1991; Dhillon et al., 1999; Whalen et al., 1999; Stahel et al., 2000). In summary, the stability of Evans blue in many of the blood-brain barrier experiments is unclear.

### (iv) tissue binding of albumin

The binding of Evans blue to albumin is an equilibrium, so an additional factor to consider is the affinity of tissues with which the dye may be exchanged from that bound to albumin (LeVeen and Fishman, 1947). This has been little investigated, but it has been shown in the aorta and common carotid arteries (Linder and Heinle, 1982) and in the lungs (Dallal and Chang, 1994) that in addition to binding to albumin a significant amount of the dye is tightly bound to the tissue. The most extensive study of tissue binding of Evans blue appears to be that of Clasen et al. (1970) which will be discussed in detail in the next section because of its particular relevance to attempts to quantitate Evans blue in brain tissue, as a measure of blood-brain barrier dysfunction.

### (v) quantitation of blood-brain barrier disruption using evans blue

The most problematic use of Evans blue in blood-brain barrier studies is in attempts to use it to quantify the magnitude of a “leak” or barrier dysfunction. This is usually done by extracting Evans blue from brain tissue at various times following intravenous or intra-arterial dye injection. Different solvents have been used to extract the dye and the amount of dye in the brain extract estimated colorimetrically, spectrophotometrically, or fluoro-spectrometrically (Evans blue exhibits natural fluorescence). There are several problems in addition to the ones discussed above concerning whether Evans blue in brain is bound to plasma proteins (and which ones) or is free and the extent to which the dye may have exchanged with brain substance or has entered cells. Colorimetric and spectrophotometric measurements are heavily dependent on the standards used because Evans blue shows spectral shifts when in protein-containing solutions. This is something that exercised physiologists considered when they were developing Evans blue for measurement of blood volume (Gregersen, 1938). These problems are likely to be worse with brain extracts because of the heterogeneity of the material compared with plasma and differences between pathological and normal brain tissue if the latter is used for standard Evans blue solutions (Hellal et al., 2004). As mentioned above many authors do not mention the composition of the standard solution used for Evans blue determinations e.g., Dhillon et al. (1999), Whalen et al. (1999), and Stahel et al. (2000). Evans blue has been suggested to be unstable in “saline” or other salt solutions, unless some protein is also present, which is why earlier workers used water as the vehicle (Gregersen and Stewart, 1938).

There is the further problem that some of the Evans blue in brain samples will also be present in contaminating blood inevitably trapped in brain capillaries. Some authors have attempted to remove blood by perfusing the circulatory system prior to removing brain samples. It is unclear how successful this is and the possibility that the dye may be washed out of the brain tissue itself does not seem to have been considered.

Estimating the size of blood-brain barrier breakdown following trauma seems particularly problematic. The initial “breakdown” is probably due to rupture of blood vessels as occurs in injury to any tissue or organ in the body, rather than dysfunction due to disruption of specific brain barrier properties (Hellal et al., 2004). No one talks about, for example, blood-muscle barrier disruption following limb trauma. There seems to be general agreement that following brain trauma there is a period of few hours when plasma proteins or other large molecules will enter the brain tissue (Habgood et al., 2007) but this probably has more to do with the time of vessel disruption and subsequent clotting of blood in the injured area of brain than actual barrier malfunction. Subsequently there is a period that may last several days when the barrier is permeable to molecules smaller than proteins (Habgood et al., 2007) as will be considered further below. The presence of clots in the vessels at the site of a brain injury makes it unlikely that a marker solution will enter the damaged region adequately. In the case of Evans blue estimates of the size of a “leak” this is likely to add to the unsatisfactory nature of attempts to quantitate the size of a barrier dysfunction using this marker.

Wang and Lai (2014) have recently published a critique of the use of Evans blue for quantitation of blood volume and blood brain barrier “leaks.” They cite some of the early literature when Evans blue was being developed for blood volume measurements and claim that Evans blue is a commonly used tracer for estimation of blood volume in humans, yet the most recent paper they cite is that of Theron and Wilson (1949). As discussed above, the use of Evans blue for that purpose was replaced by better markers many decades ago. Wang and Lai (2014) assume that Evans blue and albumin can be equated, which from the extensive discussion above about its binding to other plasma proteins and tissues, is clearly not the case. They state that it is non-toxic, which as discussed in the next section is not the case either, although toxicity depends on the amount of dye and the concentrations used. They also state that Evans blue is not taken up by cells, citing Gregersen et al. (1935). However, these authors do not make such a statement. They suggest that Evans blue may be absorbed by red cells, which might mean that it is taken up but equally that it sticks to the surface of cells. More relevant is the evidence mentioned above that Evans blue sticks to tissues, some of which may have a higher affinity for the dye than albumin. Wang and Lai (2014) cite Clasen et al. (1970) in support of their assertion that “its [Evans blue] extravasation into central and peripheral organs following a more prolonged time period correlates with vascular leakage of serum albumin, and its leakage into the brain parenchyma indicates blood-brain barrier (BBB) disruption.” Yet Clasen et al. (1970) were at great pains to explain and study experimentally the nature of binding of the dye to albumin and to tissues (and the equilibrium nature of this relationship) as well as citing evidence that Evans blue may be taken up by astrocytes. Clasen et al. (1970) estimate that as much as 15% of Evans blue in some tissues is free dye bound to tissue components, such as connective tissue. They state unequivocally that “…. the degree of coloration is not simply a reflection of the total albumin content of the tissue.”

Thus, the published estimates of the size of blood-brain barrier impairment or “leakage” are unreliable. However, some of the concerns outlined here would apply equally to other markers, particularly in the case of traumatic damage to the blood-brain barrier.

### (vi) toxicity of evans blue *in vivo*

It seems to have been little considered that Evans blue might have toxic properties *in vivo*. In a brief report Gibson and Gregersen (1935) found that rats injected with 45 mg/Kg T-1824 (Evans blue) showed decreased rate of growth and on autopsy were found to have multiple pulmonary emboli and intracellular dye in renal epithelium and hepatic parenchyma. Malaowalla and Fong (1962) injected four monkeys with different doses of Evans blue. One receiving 25 mg/Kg survived and was apparently normal. The other three injected with 50, 100, or 200 mg/Kg died within days of injection. The only systematic study appears to be that of Hueper and Ichniowski (1944) who reported on the long term toxic effects of intravenously administration of different dose levels in dogs, cats, rabbits and rats. Aqueous solutions of 0.5% were used, which is in fact a lower concentration than generally used in blood brain barrier experiments (2%). Rats were given 1, 3, 5, or 10 ml/Kg injections of the dye. Three of ten animals in the two larger dose groups died at 1–3 months, with widespread pathological changes in the brain, lungs, heart and liver. Other animals were killed at 6 months; about half of those examined showed substantial degenerative lesions in the epithelium of the seminiferous tubules. This was examined in shorter-term experiments in which 25 rats were administered two injections of 1 ml of a 1% aqueous solution of Evans blue with 3 days between injections. Fourteen rats died within 14 days, the survivors were killed and all animals examined histologically. Nearly 50% of the 25 animals showed testicular degeneration to varying degrees. Other organs were not commented upon, but it seems unlikely that the animals would have died from a sole pathology of testicular degeneration, suggesting other severe pathology in response to the Evans blue injections. Many blood-brain barrier experiments employ 4–5 ml/Kg Evans blue solutions in concentrations of 2% (e.g., Uyama et al., 1988; Dhillon et al., 1994; Whalen et al., 1999; Kaya and Ahishali, 2011; Manaenko et al., 2011) but sometimes as high as 4% (Jiang et al., 2014). The duration of the experiments is generally much less than in Hueper and Ichniowski (1944), in the range of 10 min to 24 h. So it is unclear whether there would have been histologically detectable lesions within that time span. There appear to be no reports on this, apart from the observations of Moos and Møllgård (1993) from which they concluded that the Evans blue they could detect in brain a few hours after injecting 2% Evans blue into mice might be due to toxic effects on cerebral endothelial or ependymal cells; this suggests that at the most commonly used concentration of Evans blue (2%) there may well have been toxic effects.

Thus, there is good reason to be concerned about possible toxic effects of Evans blue in short-term experiments involving the concentration of dye most commonly used (2%).

*In summary*, it seems that all of the frequently asserted properties of Evans blue as an exclusive marker for plasma albumin are sufficiently suspect as to make it unsuitable for use in studies of blood-brain barrier integrity. Evans blue detected in brain is likely to be a mixture of dye bound to plasma proteins (which ones and the quantitative extent depending upon the species), dye bound to brain tissue and free dye. This point does not seem to have been specifically studied in the brain, but as mentioned above, has been examined in the lungs, following systemic injection of Evans blue dye (Dallal and Chang, 1994). There are other markers, which do not suffer from these problems. It seems baffling that Evans blue continues to be used so widely.

## Horseradish peroxidase in assessment of brain barrier permeability

The first mention of horseradish peroxidase (HRP) exclusion from the brain after injection of a solution into the circulation appears to be that of Straus (1959) although he is rarely credited with this observation. The introduction of HRP into blood-brain barrier studies was an important technical achievement, which led to significant advances in understanding blood-brain barrier biology. Of particular note are the papers by Karnovsky (1967), Reese and Karnovsky (1967), and Brightman and Reese (1969). The importance of the introduction of HRP is that the reaction product of this peroxidase can be made electron-dense, so it is possible to visualize it at the electron microscopical level. The paper of Brightman and Reese (1969) involved both intravascular and intrathecal injection experiments. This milestone paper established the ultrastructural basis for the barrier at the cerebrovascular interface between blood and brain. These detailed studies showed that the primary barrier was the intercellular tight junction between adjacent endothelial cells although an additional barrier feature was the paucity of intracellular pinocytotic vesicles in the endothelial cells. They also showed tight junctions between the epithelial cells of the choroid plexus providing a barrier to HRP at the blood-CSF interface. The experiments described in these papers (Karnovsky, 1967; Reese and Karnovsky, 1967; Brightman and Reese, 1969) were all performed in mice, with no reports of HRP toxicity.

However, when different strains of rats (and also guinea pigs) were used subsequently, some technical problems with HRP became apparent. These were well understood by the people who developed the use of HRP for permeability studies in various tissues, most notably Graham and Karnovsky (1966) and Cotran and Karnovsky (1967, 1968). It was demonstrated that HRP can cause degranulation of mast cells with the release of histamine and serotonin, which have been shown to affect vascular permeability (Majno et al., 1961) in some commonly used strains of rats (Cotran and Karnovsky, 1967) but not in others (Cotran et al., 1968). In early studies of barrier permeability these rat strains (e.g., Sprague-Dawley) were treated with anti-histamine and anti-serotonergic agents (Cotran and Karnovsky, 1968). However, in later studies this precaution seems to have been overlooked by some (e.g., Farrell and Shivers, 1984; Lotocki et al., 2009; Kaya and Ahishali, 2011; Cunningham et al., 2014) or only antihistamines were used (Tanno et al., 1992; Dietrich et al., 1994; Ueno et al., 2002). In other studies the problem was avoided by using Wistar rats (Ugrumov et al., 1983; Dietrich et al., 1993; Cevik et al., 2013) in which HRP has been reported not to produce mast cell degranulation (Cotran et al., 1968) although it is unclear whether Wistar rats were chosen with this problem in mind. Given these studies were aimed at evaluating blood-brain barrier permeability, in studies in which no inhibitors were used in strains of rats that are known to be sensitive to HRP, this throws the value of results from such experiments in doubt. Even in mice deleterious effects of HRP have been demonstrated, but they appear to be dose dependent and possibly also on the type of HRP used. Thus, Clementi (1970) showed that doses of 24 mg/100 g in Swiss albino mice affected lung capillary permeability, but not when 1 mg/100 g was used. Also in Wistar rats 5–10 mg/100 g body weight HRP (but not 1 mg/100 g) resulted in profound hypotension due to histamine release which could be inhibited by pre-treatment with the antihistamine promethazine (Deimann et al., 1976). A wide range of HRP doses has been used in both rats and mice, ranging from 0.4 mg/100 g body weight in Sprague–Dawley rats (Cotran and Karnovsky, 1968) to 33 mg/100 g in adult Wistar rats (Pluta et al., 1994), 90 mg/100 g in postnatal Wistar rats (Ugrumov et al., 1983) and 20–40 mg/100 g in adult white mice (Brightman and Reese, 1969).

Ototoxicity of HRP has been described in guinea pigs (Ross et al., 1977). There is also evidence of anomalous permeability results when comparing HRP with other barrier permeability markers, which, as was suggested, could be due to membrane damage (Mazariegos et al., 1984).

We suggest that an additional factor, which does not seem to have been considered, is the possibility that anesthesia might modify or mask the toxic responses to HRP, although we are not aware of any evidence for this. Some of the early experiments did not involve anesthesia because they were confined to tail vein injections (e.g., Brightman and Reese, 1969), but later experiments involved a range of volatile and injected anesthetic agents. In addition, it often seems not to be appreciated that what is visualized in electron microscopy of HRP-containing tissues is the reaction product of the peroxidase, not the protein itself. Which raises the possibility of diffusion artifacts resulting in a distribution of the reaction product that may not reflect that of the actual protein.

Overall it seems reasonable to be cautious about interpreting the results of experiments using HRP, especially when large doses were employed and there was no pre-treatment with antihistamines.

## More appropriate markers of blood-brain barrier dysfunction

### Radiolabeled markers

The use of radiolabeled sucrose and inulin for blood-brain barrier permeability studies was pioneered by three giants in the blood-brain barrier field: Dixon Woodbury, Hugh Davson and Bill Oldendorf (e.g., Reed and Woodbury, 1963; Reed et al., 1964; Davson and Bradbury, 1965; Davson and Oldendorf, 1967; Oldendorf and Davson, 1967; Davson and Segal, 1969, 1996). An important advantage of these markers is that they allow a quantitative determination of blood-brain or blood-CSF permeability. However, experiments in which they are used need to be designed carefully if spurious results are not to be obtained. This includes (i) ensuring that steady-state plasma levels of maker are achieved, (ii) that blood contamination of brain samples by the marker is estimated and (iii) the retention of the isotopic label on the marker is secure.

Sucrose is a small water-soluble molecule that is not metabolized to a significant extent if injected parenterally. It distributes in the extracellular space of most tissues and organs of the body, but because of the limited permeability of the blood-brain and blood-CSF barriers and the turnover of CSF, sucrose does not reach a concentration in brain that reflects the true extracellular space. This is a phenomenon Davson described as the “sink effect” (see Davson and Segal, 1996). A better estimate of brain extracellular space using labeled sucrose has been obtained by a combination of intravascular administration and perfusion through the ventricular system (Oldendorf and Davson, 1967). Because sucrose is excreted via the kidneys, a single injection causes a rapid rise in blood level, followed by a rapid decline with mixing and distribution into the extracellular fluid and then a slower but steady decline due to loss in the urine. This means that unless measures are taken to maintain a steady-state plasma level of marker results expressed as a ratio brain/plasma or CSF/plasma, then the falling plasma level will give rise to a spurious finding that these ratios increase over the period of an experiment in some cases up to 24 h (e.g., Ferguson and Woodbury, 1969). As described in detail by Davson in many papers and text books (e.g., Davson and Segal, 1996) an approximately steady state level in plasma can be obtained by nephrectomy and either continuous infusion or intermittent injection of sucrose during the course of the experiment. Plasma samples collected throughout the experiment can then be used to calculate a time-weighted mean plasma concentration for calculation of a ratio based on terminal brain and CSF samples. Although nephrectomy might appear to be a severe and unphysiological intervention, in short term experiments lasting only a few hours it is probably not affecting the results of such experiments. In fetal sheep, nephrectomy is unnecessary as the kidneys are not yet producing much urine but similar experiments with radiolabeled sucrose have been obtained (Evans et al., 1974). With respect to CSF samples it is essential that the samples are checked for blood contamination (see Habgood et al., 1992). For brain samples an estimate of blood contamination is important otherwise ratios will be inflated by the presence of marker in blood within the brain sample. This can be achieved by injecting a second marker a few minutes before the end of an experiment. It is necessary for this marker be present in the circulation long enough to mix properly but not to penetrate into the brain to any measurable extent. Radiolabeled markers such as ^113m^Indium, which binds to transferrin and has the advantage of a very short half-life, have been used for this purpose (Evans et al., 1974) but other markers such as radiolabeled albumin or inulin would also be suitable. Some authors have attempted to deal with the problem of blood contamination of brain samples by perfusing the brain with some form of “physiological” solution prior to removing the brain. It is not clear how successful this is, particularly in experiments involving brain trauma where part of the cerebral circulation will be obstructed by post trauma blood coagulation within vessels. Care is also required to make sure the radiolabel is exclusive to the marker in use and remains attached to the marker molecule throughout the experiment. Providing these factors are taken into consideration the use of radiolabeled markers is a valuable way of obtaining a quantitative estimate of any brain barrier dysfunction. Their disadvantage is that the radiolabel cannot be visualized in tissue sections to a satisfactory level of resolution, so the morphological nature of the dysfunction cannot be ascertained. This problem has been overcome by the use of biotin labeled dextrans (see below).

### Endogenous and exogenous plasma proteins

Immunohistochemical detection in brain sections of proteins in plasma such as albumin (e.g., Dziegielewska et al., 1991; Liddelow et al., 2009; O'shea et al., 2014) immunoglobulin (e.g., Garbuzova-Davis et al., 2012; O'shea et al., 2014; Blair et al., 2015) and fibrinogen (e.g., Bridges et al., 2014) has been used to visualize breaches in the blood-brain barrier. Endogenous proteins have the advantage that they are already present *in situ* and do not need to be injected thus avoiding potentially un-physiological conditions. Their limitation is that once they have entered the extracellular space of the brain they will continue to diffuse and therefore are not a reliable index of the duration or progression of the leak across brain barriers (Habgood et al., 2007). This problem can be circumvented by injecting an exogenous protein and detecting it with an antibody that does not cross-react with the native protein or a fluorophor-labeled albumin (Dziegielewska et al., 1991; Pelz et al., 2013; Lehmann et al., 2014; Krueger et al., 2015). As will be discussed below this method and any other involving visualization of a marker at the light microscopical level, may be of insufficient resolution to determine the cellular nature of the barrier disruption, although it is common for dysfunction to be equated with disruption of tight junctions (Jin et al., 2014). Equally changes in immunostaining of tight junction proteins (often erroneously described as changes in expression e.g., Lucke-Wold et al., 2014) are often confused with changes in permeability. These conclusions can only be reached if appropriate ultrastructural studies are carried out in parallel. As will be discussed below, this is rarely the case.

### Sodium fluorescein

Sodium fluorescein (376 Da) was the first visualizable small molecular sized marker to be introduced into the blood-brain barrier field (Hoffman and Olszewski, 1961; Malmgren and Olsson, 1980). Unlike the more commonly used dyes such as Evans blue, sodium fluorescein binds only weakly to proteins and appears to be an effective low molecular weight marker for brain barrier studies in contrast to dyes that bind to proteins (Wolman et al., 1981). Kaya and Ahishali (2011) have suggested that spectrophotofluorimetric sodium fluorescein uptake measurements (excitation at 440 nm and emission at 525 nm) may enable detection of more subtle alterations in blood-brain barrier permeability when compared to the use of radioactive tracers and may thus be a sensitive indicator of early stages of barrier permeability. No specific effects of sodium fluorescein on blood-brain barrier appear to have been reported. The LD50 in mice was estimated as 4738 ± 1.23 mg/Kg body weight (Yankell and Loux, 1977) whereas the amount injected into mice for barrier permeability experiments was only a fraction of this, for example 50 mg/Kg body weight (Malmgren and Olsson, 1980). A single dose of 500 mg/Kg in pregnant mice was reported not to have any embryotoxic or teratogenic effects (Salem et al., 1979).

Thus, sodium fluorescein appears to be considerably less toxic than Evans blue or HRP and unlike Evans blue shows only weak binding to proteins in plasma, which given some justification for it being the second (with HRP) most commonly used marker for blood-brain barrier integrity (Figure 2).

### Dextrans

These are complex branched polysaccharides made of many glucose molecules. They consist of chains of lengths varying from 3 to 2000 kDa. They are commercially available labeled either with a fluorophor or biotin. An even smaller biotin labeled molecule, ethylenediamine (286 Da) is also available. This is smaller than sucrose (342 Da), which is a commonly used marker for quantitative studies of brain barrier permeability. There were early reports of dextran toxicity when injected into paws of rats due to histamine and 5-hydroxytrypatamine release that could be attenuated by the use of specific inhibitors (Rowley and Benditt, 1956). There has been quite extensive research on dextrans as potential plasma expanders in trauma cases or other circumstances of substantial blood loss. Proper discussion of this is outside the scope of this review. Suffice it to say that the use of dextrans clinically is limited by a significant incidence of anaphylactoid reactions (Lundsgaard-Hansen, 1969; Medby, 2014) and coagulopathy due to interference with some of the clotting factors (Hahn, 2013; Medby, 2014) although this property has been put to good use in prevention and treatment of deep vein thrombosis (Medby, 2014). In animal experiments it was shown that release of histamine and 5-hydroxytryptamine occurs from mast cell degranulation as also reported for HRP (see above). In rabbits administered intravenous dextran preparations over several weeks, there were toxic effects following daily doses of 20 mg/Kg body weight, but not with 10 mg/Kg (Hint and Richter, 1958). There are reports that the anaphylactoid effect of dextrans depended on the preparation and is less in smaller molecular weight preparations (Edlund et al., 1952; Walton, 1954); it is unclear to what extent the effects may have been due to contaminants. The biotin and fluorophor labeled dextrans now in experimental use are highly purified and because of the sensitivity of the techniques applied to visualize the labels, only small amounts are required. In our experience we have not seen any untoward effects *in vivo* (e.g., Ek et al., 2001, 2003).

**Figure 5:**
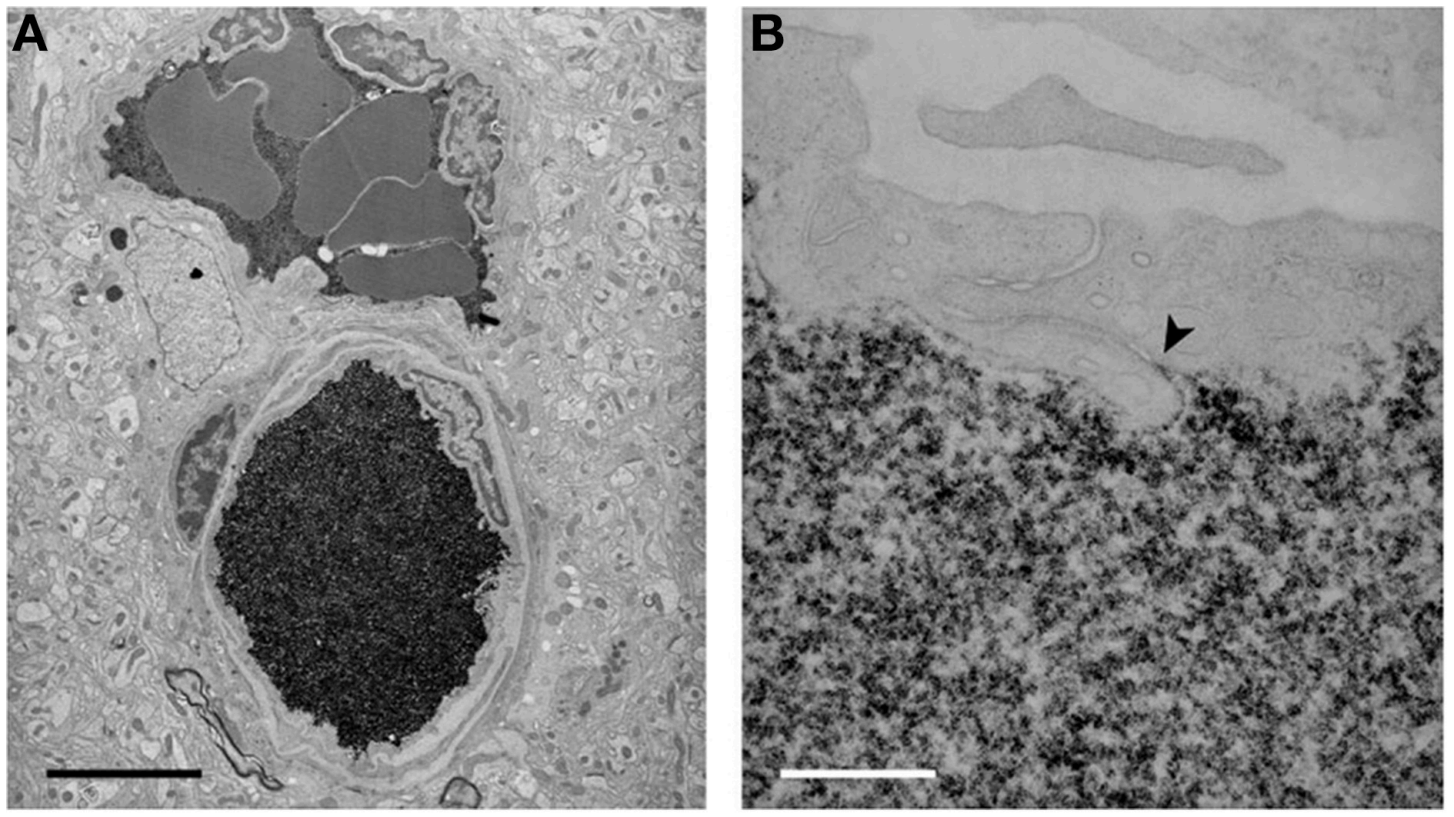
**Electron micrographs of the localization of biotin ethylenediamine (BED) in blood vessels deep inside the cortex of a 2-month-old opossum 10 min after an intravenous injection**. Similar staining is found after an intravenous injection of biotin-dextran (BDA3000). **(A)** Low-power micrograph showing two paired vessels with abundant reaction product within lumen. No reaction product is visible in the surrounding tissue. Pairs of arteries and veins are characteristic of the vascular pattern in marsupial brains (Wislocki and Campbell, 1937). **(B)** High-power micrograph of an interendothelial cleft showing that the tight junctions in the young adult restrict the passage of BED through the cleft (arrowhead). Scale bar = 4 μm in **(A)**; 300 nm in **(B)**. From Ek et al. (2006).

**Figure 6:**
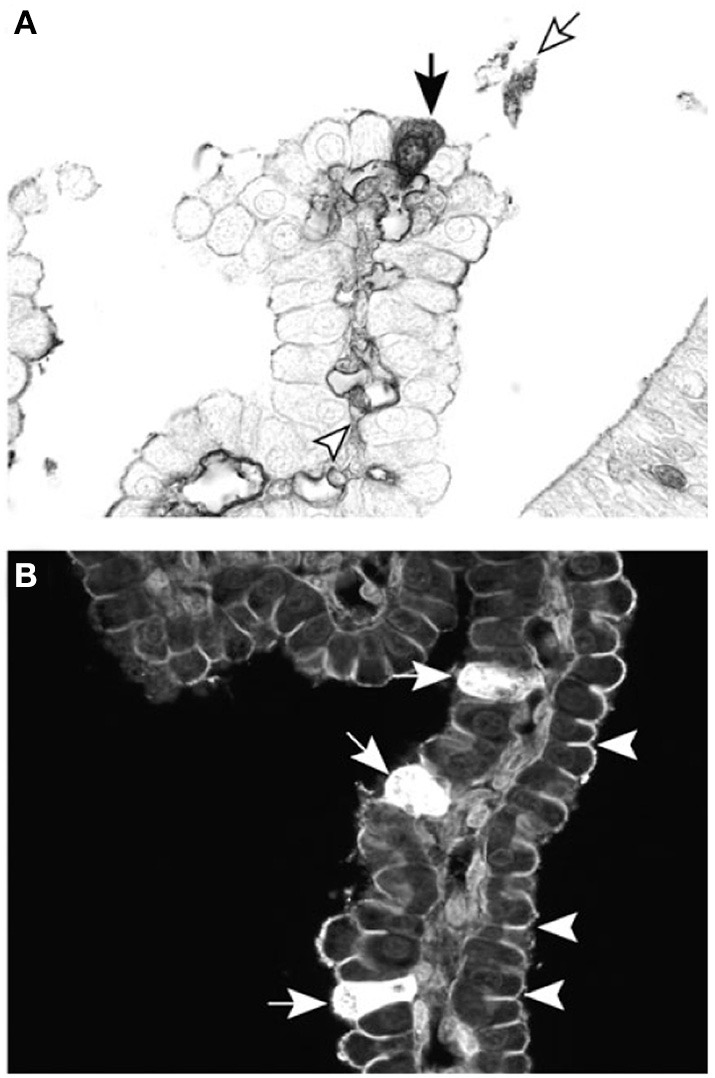
**Cellular localization of dextran probes in postnatal lateral ventricular choroid plexus of the marsupial South American opossum (*Monodelphis domestica*)**. **(A)** Forty-five minutes after intraperitoneal injection with BDA-3 kDa, the probe can be seen in individual epithelial cells of the choroid plexus (filled arrow), as well as in the blood vessel lumen (arrowhead) and precipitated in the CSF (unfilled arrow). **(B)** Ten minutes after intraventricular injection with BDA-3 kDa–Fluorescein, more epithelial cells take up the probe (filled arrows) following CSF injection compared with intraperitoneal injection **(A)**. Penetration of the fluorescent probe between epithelial cells is stopped by the presence of tight junctions (examples highlighted by arrowheads). Scale: 50 μm. From Liddelow et al. (2009).

Biotin labeled molecules can be visualized both at the light microscopical and electron microscopical level (Ek et al., 2003, 2006; Johansson et al., 2006) as illustrated in Figure 5. The blood-CSF permeability characteristics of a range of different sized biotin labeled dextrans (and biotin ethylenediamine) have been shown in quantitative studies to be similar to the permeability of more traditional permeability markers, L-glucose, sucrose and inulin and to be consistent with diffusion across the blood-CSF interface (Ek et al., 2001, 2006). Use of fluorophor and biotin labeled dextrans led to the unexpected finding that in the developing and adult choroid plexus the route of entry from blood to CSF is an intracellular one via the plexus epithelial cells (Ek et al., 2003; Liddelow et al., 2009), as illustrated in Figure 6 rather than intercellular via the tight junctions as generally believed (Anderson, 2001; Nitta et al., 2003; Piontek et al., 2008; Abbott et al., 2010).

*In summary*, labeled dextrans are valuable markers of blood-brain barrier integrity, which in the small concentrations used appear to be non-toxic. The biotin labeled form is particularly valuable as it can be visualized at both the light and electron microscopical level. Also they can be quantitated in CSF (Ek et al., 2006).

## Blood-brain barrier dysfunction in neurological disorders

In the past 10–15 years there has been a resurgence of interest in the possibility that the disruption or malfunction of the blood-brain barrier may be involved in a wide range of neurological disorders: (Keep et al., 2014) including dementia (van de Haar et al., 2015) Alzheimer's disease (Provias and Jeynes, 2014), multiple sclerosis (Lengfeld et al., 2014; Kamphuis et al., 2015), stroke (Schoknecht et al., 2014; Cui et al., 2015), diabetes (Liu and Liu, 2014; Prasad et al., 2014) and trauma to the brain (Barzó et al., 1997; Whalen et al., 1998; Stahel et al., 2000; Habgood et al., 2007) and spinal cord (Oudega, 2012; Figley et al., 2014). However, it is often not clear whether barrier dysfunction is involved in the primary pathology or as a consequence of the pathology, perhaps exacerbating the effects of the disorder. There is generally a rather simple assumption that the barrier may be breached, rather than considering the nature of the breach or the possibility that the many cellular transport mechanisms (in and out of the brain) might be affected. It is perhaps the simple view of the blood-brain barrier as a mere mechanical barrier that has led to such widespread use of Evans blue, without any realization of its limitations as discussed above or the nature of the functional mechanisms that may be affected.

More recent studies are beginning to examine specific barrier mechanisms in neurological disorders, for example Cui et al. (2015) claimed to demonstrate that ATP-binding cassette, ABC1 (cholesterol efflux pump) deficiency in stroke induced in mice increased blood-brain barrier leakage of immunohistochemically detected extravascular albumin. However, what they actually showed was an association between ABCA1 deficiency and albumin leakage; as they did not do high-resolution microscopy they did not demonstrate the pathway across which the albumin leakage occurred. More importantly there are likely to have been many other transporter changes consequent upon the ischaemic injury, which could have contributed to the barrier dysfunction. The importance of this study is the demonstration of a change in a transporter, rather than a leakage of protein, which is already well known to occur in stroke (Krueger et al., 2015). Liu and Liu (2014) have summarized studies showing that a number of ABC transporters change their expression levels in blood vessels of brains of rats with induced diabetes, but there are differences in regulation that depend both on the specific transporter and on the brain region. A quite different disorder of blood-brain barrier function is heterozygous mutation in the GLUT1/SLC2A1 gene, resulting in GLUT1-deficiency syndrome (GLUT1-DS), which manifests itself in infants as motor and mental developmental delay, seizures, reduced head growth and a movement disorder with ataxia, dystonia, and spasticity (Brockmann, 2009).

The study of Krueger et al. (2015) is particularly important because it is one of the few to employ immunohistochemistry of tight junction proteins and electron microscopy of the distribution of a barrier marker (FITC-albumin). They showed that there was no change in either the tight junction proteins or the ultrastructure of the junctions post stroke in adult rats. They demonstrated that disruption of the blood-brain barrier resulted in FITC-albumin-containing transendothelial vesicle trafficking and signs of degeneration. These findings make it clear that the common assumption that a leakage of marker (often Evans blue) and/or down regulation of tight junction proteins are not synonymous with disruption of tight junctions. For this to be established requires ultrastructural studies using suitable electron dense permeability markers or ones that can be rendered electron dense by suitable processing (Ek et al., 2003, 2006).

## Conclusions

The properties, advantages and disadvantages of the various markers discussed above are summarized in Table 2. For the many reasons outlined in this review, Evans blue is an unsatisfactory tool for studying blood-brain barrier dysfunction. The most that can be said in its favor is that it may be useful in some circumstances as a rapid and inexpensive method for checking for the presence of a barrier leak. But for proper understanding of a barrier disruption there are now much better methods available and its seems reasonable to propose that Evans blue has had its day and is long overdue to be pensioned off from brain barrier studies as happened long ago for its use for blood volume estimates.

**Table 2:** **Characteristics of blood-brain barrier markers described in this review**.

**Marker**	**Size**		**Binding^1^**		**Visualization**			**Quantification^3^^,^^4^**	**Toxic^5^**	**Renal clearance^6^**	**Cost**
	**Da**	**Radius nm**	**Protein**	**Tissue^2^**	**Macro**	**LM**	**EM**				
Biotin ethylenediamine	286^a^	NR	NR	No	No	Yes	Yes	Qualitative only	NR	Yes	Med
Radio-sucrose	342.3^b^	0.51^i^	No	No	No	No	No	Accurate	No	Yes	High
Na fluorescein	376^c^	NR	Weak	NR	No	Yes	No	Unreliable	No	Yes	Low
Evans blue	960^d^	NR	Yes	Yes	Yes	Yes	No	Unreliable	Yes	No^*^	Low
Trypan blue	961^e^	NR	No	Yes	Yes	Yes	No	Unreliable	Yes	No^*^	Low
Radio-inulin	≈7000^b^	1.3^i^	No	No	No	No	No	Accurate	No	Yes	High
Horseradish peroxidase	≈44,000^f^	3.0^j^	NR	NR	No	Yes	Yes	Unreliable	Yes	No	Low
Albumin (unlabeled)	69,000^g^	3.5^i^	No	No	No	IHC	Yes	Unreliable	No	No	Low
Radio-albumin	69,000^g^	3.5^i^	No	No	No	IHC	No	Accurate	No	No	High
IgG	≈155,000^g^	5.3^i^	No	No	No	IHC	No	Qualitative only	No	No	Low
Fibrinogen	340,000^g^	11.0^k^	No	No	No	IHC	No	Qualitative only	No	No	Low
Dextrans	1500 to 2,000,000^h^	0.8–38.2^l^	NR	NR	No	Yes	Yes	Qualitative in tissue	No	Only low MW	High

MW, molecular weight; Macro, visible to unaided eye; LM, light microscopy; EM, electron microscopy; NR, not reported; IHC, immunohistochemistry; Med, medium cost;

* unless protein binding capacity in plasma exceeded.

a Ek et al. (2006),

b Weast (1986),

c Malmgren and Olsson (1980),

d Allen and Orahovats (1950),

e T1826, isomer of Evans blue, Allen and Orahovats (1950),

f www.sigmaaldrich.com,

g Thompson (2005),

h http://www.thermofisher.com,

i Dziegielewska et al. (1979),

j Rennke et al. (1978),

k Boyd et al. (1969),

l Armstrong et al. (2004), Grznárová et al. (2005).

1 Dyes are generally problematic because they bind to proteins in plasma and to tissues in a reversible equilibrium, so when visualized it is unclear whether they are free dye or bound dye and how they became located at a particular site.

2 Many markers may be taken up by a variety of cells, including choroid plexus epithelial cells. Also neurons and glia, but only if the blood-brain barrier is breached.

3 With currently available methods only markers with radiolabels (e.g., ^3^H, ^14^C, ^125^I) can be reliably quantitated. For reliable measurements this requires checking on the stability of the label and absence of contaminating labeled products (Evans et al., 1974). For liquid scintillation counting of ^3^H and ^14^C particular care is required to allow for differential quenching from variations in protein content of samples; internal quench correction is usually inadequate. Colorimetric and spectrophotometric methods are generally inaccurate because of spectral shifts produced by different composition of brain tissue and standards.

4 Many groups attempt to deal with the problem of blood contamination by perfusing the cerebral circulation with “physiological” solutions at the termination of the experiment; this is probably variably effective, particularly in the case of any local disturbance to cerebral circulation e.g., brain trauma, as local intravascular coagulation will limit entry of both marker and of washout fluid.

5 At concentrations used in marker experiments.

6 Depends on molecular diameter, charge and protein binding. Dyes that bind to proteins in plasma would not be expected to appear in urine unless the concentration in plasma exceeded the binding capacity of the proteins. Neutral substances with effective molecular diameters of < 4 nm are freely filtered. Above 8 nm filtration is negligible. Between these values the amount filtered depends on molecular diameter and charge (Barrett et al., 2012).

## Author contributions

All of the listed authors contributed to the conception, design, research, drafting, and final approval of the work. They each agree to be accountable for all aspects of the work.

### Conflict of interest statement

The authors declare that the research was conducted in the absence of any commercial or financial relationships that could be construed as a potential conflict of interest.

## Abbreviations

CNS: central nervous system
CSF: cerebrospinal fluid
HRP: horseradish peroxidase.
